# Isolation of New Chemical Modulators of the Interaction Between HIV-1 Integrase and the Cellular Restriction Factor GCN2

**DOI:** 10.3390/v17081138

**Published:** 2025-08-20

**Authors:** Chloé Torres, Floriane Lagadec, Eugenia Basyuk, Patricia Recordon-Pinson, Mathieu Métifiot

**Affiliations:** Andevir, University Bordeaux, CNRS, Microbiologie Fondamentale et Pathogénicité, UMR 5234, F-33000 Bordeaux, France; chloe.torres@u-bordeaux.fr (C.T.); floriane.lagadec@u-bordeaux.fr (F.L.); eugenia.basyuk@u-bordeaux.fr (E.B.); patricia.recordon-pinson@u-bordeaux.fr (P.R.-P.)

**Keywords:** protein–protein interaction, HIV-1 integrase, integrated stress response (ISR), GCN2, AlphaScreen, structure–activity relationship (SAR), high-throughput screening (HTS)

## Abstract

Integrase is a key protein during HIV-1 replication as it catalyzes the integration of viral DNA into the host DNA. After several decades of research, highly potent and selective active site inhibitors have emerged. The new challenge is now to develop molecules with an original mode of action, targeting integrase out of its catalytic site. During a previous study, we developed an in vitro assay to monitor the interaction between HIV-1 integrase and one of its cellular partners, GCN2. This AlphaLISA-based assay was validated as a platform for chemical modulator screening. In the present study, we used a library of natural products from the Developmental Therapeutics Program (NIH) to identify novel chemical leads. The best modulators were characterized and a structure–activity relationship study was initiated with a limited number of derivatives. We found that most inhibitors were tricylic or tetraclyclic molecules, with the most potent belonging to the anthracyclines/anthraquinones. Of note, several molecules exhibited interesting cellular activities and may be suitable for further optimization.

## 1. Introduction

Since the discovery of HIV in 1983, antiretroviral therapy (ART) has undergone tremendous evolutions [1]. The first drug approved for AIDS was AZT (zidovudine) in 1987, a nucleoside analog targeting reverse transcriptase (RT). However, shortly after its release, it faced the rapid appearance of resistance. Only a combination of drugs acting on different targets may raise the genetic barrier to resistance, with the drawback of additional side effects in patients. This challenge was finally overcome by the development of non-nucleoside RT inhibitors and other molecules targeting the two other viral enzymes protease (PR) and integrase (IN) [2]. Since then, the arsenal of therapeutics has continued to be optimized, using new targeting strategies and administration modes to facilitate patient treatment and adherence [3,4]. However, natural polymorphism, the selection pressure of antiretroviral therapy, and, in some countries, inadequate follow-up of patients have led to the emergence of resistance mutations even with the most recent agents (e.g., dolutegravir, lenacapavir) [5,6].

Of the three virally encoded enzymes, HIV-1 IN has proven to be the most difficult to target [7]. This protein belongs to the polynucleotidyl transferase family and is structurally related to human RNase H1, which raises concerns about its “drugability” without toxicity. IN is a 32 kDa protein (288 amino acids) composed of three domains, all involved in DNA binding and oligomerization of the enzyme. The core domain (50–212) harbors a catalytic triad DDE that binds two metal cofactors (Mg^2+^) responsible for the two successive transesterification reactions needed to perform integration. After binding the viral DNA long terminal repeats (LTRs), the terminal dinucleotide at the 3′ ends are cleaved (3′-processing, 3′-P). Then, these newly created 3′-OH ends are used to attack the target DNA (strand transfer, ST), leading to the integration of the provirus into the host genome. Nonetheless, a post-integration repair is required to restore DNA integrity, and this is supported by cellular processes (5′ overhang recession and gap filling).

From a therapeutic point of view, IN has been validated as a target by the introduction of raltegravir (RAL) to the clinic in 2007. This first-in-class drug is a highly specific catalytic inhibitor that binds at the interface of the enzyme and the DNA substrate, chelating the two metal cations in the active site [8]. Because RAL and other IN active site inhibitors exhibit a relative selectivity for the inhibition of ST over 3′-P, this class of molecules is often referred to as the IN strand transfer inhibitors (INSTIs). However, resistance to INSTIs arose with mutations in the IN-coding region, leading to a reduced susceptibility to this drug class. Accordingly, a new class of molecules targeting IN out of its active site has been in development to circumvent this problem.

Besides its canonical role in integration, IN has non-catalytic functions during replication [9,10]. It has been implicated in (i) the reverse transcription step thanks to its interaction with RT, (ii) nuclear translocation of the pre-integration complex (PIC) through its nuclear localization signal (NLS) and interaction with nuclear pore complex proteins and transport-associated proteins, and (iii) virus packaging and morphology, including interactions with the viral RNA and capsid. Altogether, this emphasizes the crucial role of IN during HIV replication, not only because of its catalytic activity but also through its numerous protein–protein interactions. Among them, the most described partner of IN is the transcription factor lens epithelium-derived growth factor (LEDGF)/p75 [11]. LEDGF interacts with IN (core domain) through its integrase binding domain (amino acid residues 347-429) at a dimer interface, promoting IN oligomerization. Because of its chromatin binding activity, LEDGF tethers IN and subsequently the PIC to the integration site [12,13]. Altogether, the cellular integration activity is highly dependent on the presence of LEDGF and knocking down or knocking out the protein resulted in a dramatic reduction in integration efficacy and viral replication. Accordingly, molecules targeting the LEDGF binding site of IN were developed [14]. Unexpectedly at the time, targeting the LEDGF binding site of IN resulted in an allosteric inhibition of IN catalytic activities and induced morphologic aberrations of the newly produced viral particles [15,16]. Accordingly, these molecules are referred to as LEDGINs (LEDGF-IN inhibitors) or ALLINIs (Allosteric IN inhibitors). Still, new hope was raised by the development of SPT0404. This pyrrolopyridine derivative has entered phase 2a clinical trials (NCT05869643). In vitro, it promotes IN multimerization and inhibits RNA and LEDGF binding, resulting in a low nanomolar antiviral activity [17,18].

In parallel, other protein–protein interactions have been explored. Among them, IN interacts with Ku70, which is part of the DNA-dependent protein kinase (DNA-PK) complex and involved in DNA damage repair (non-homologous end Joining, NHEJ) [19]. A virtual screening enabled the identification of a novel series of compounds based on the pyrrolo [1,2-a]quinolone scaffold [20]. Unfortunately, while the lead compound (s17) is able to inhibit the IN-Ku70 interaction with an IC_50_ of 13 ± 2 µM, none of the derivatives tested to date exhibited enhanced activity. Still, Anisenko and collaborators were able to show that s17 was antiviral at a similar concentration without interfering with NHEJ in cells. Altogether, there have been several examples of the successful targeting of a protein–protein interaction interface to inhibit HIV-1 IN, which makes this strategy a promising avenue for the development of novel anti-HIV inhibitors.

In a previous study, we identified GCN2 (general control nonderepressible 2) as a cellular partner of IN [21]. GCN2 is one of the four eIF2α kinases involved in the integrated stress response (ISR) [22]. HIV-1 infection activates GCN2 (autophosphorylation) leading to a transient translation arrest in the cell [23,24]. In parallel, we showed that GCN2 not only interacts with IN but also phosphorylates the enzyme on its C-terminal domain [25]. Mutation of residue S255 to alanine, preventing its phosphorylation, resulted in an increase in viral DNA integration in cells. Conversely, GCN2-depleted cells displayed higher levels of replication, which is in line with a restrictive role of GCN2 on HIV replication. Therefore, a small molecule able to stabilize the IN-GCN2 interaction may serve as a novel inhibitor of HIV replication. Nonetheless, altering the assembly/disassembly dynamics of the PIC can have unexpected consequences, as seen with ALLINIs. Indeed, GCN2 not only affects IN activities through the phosphorylation of the S255 residue but interacts with the IN-LEDGF complex. Thus, GCN2 could also play a role as a scaffolding protein. Accordingly, small molecule modulators of the IN-GCN2 interaction, either inhibitors or stimulators, may affect HIV replication.

After setting up an in vitro interaction assay based on AlphaLISA technology, a pilot screen using 133 FDA-approved drugs (DTP oncology set) was conducted to identify modulators of the IN-GCN2 interaction [26]. In the present study, we performed a new screen with a larger, more diverse library of molecules (DTP Natural Products Set, 420 molecules). The best compounds from the two experiments were selected for further structure–activity relationship studies. Altogether, we identified five new chemical scaffolds that inhibited the IN-GCN2 interaction in the low micromolar to high nanomolar range. Among them, we identified compounds with antiviral activity in the mid-nanomolar concentration range, as well as compounds able to stimulate HIV replication.

## 2. Materials and Methods

### 2.1. Chemicals

The Approved Oncology Drugs Set VIII (133 molecules), the Natural Products Set V (420 molecules), and the individual molecules used in this study were obtained through the National Cancer Institute Developmental Therapeutics Program (NCI DTP, NIH). Molecules were provided either as 10 mM stock solutions in 100% dimethylsulfoxide (DMSO) or as powders that were dissolved the same way and stored at −20 °C.

### 2.2. Oligonucleotides

The short oligonucleotides 19T (GTGTGGAAAATCTCTAGCA) and 21B (ACTGCTAGAGATTTTCCACAC) were purchased from Integrated DNA Technologies, Inc. (Leuven, Belgium) and PAGE-purified in-house. IN ST substrate was generated by annealing 3′ radiolabeled 19T to 21B as previously described [27].

### 2.3. Proteins

GCN2 was commercially available from SignalChem (Richmond, Canada; EIF2AK4 (GCN2), cat. #E12-11G). It is an active truncated version of the human protein (192-1024) fused with an N-terminal glutathione S-transferase (GST) tag for a molecular weight of about 132 kDa. Stock solutions were provided at a concentration of 0.1 mg/mL in storage buffer (50 mM Tris-HCl pH 7.5; 150 mM NaCl; 10 mM glutathione; 0.1 mM EDTA; 0.25 mM dithiothreitol (DTT); 0.1 mM phenylmethanesulfonyl (PMSF); 25% glycerol) and stored at −80 °C.

Recombinant full-length HIV-1 IN was produced in-house as previously described [27]. Briefly, expression of IN was obtained by transformation of BL21(DE3)pLysS (Invitrogen) with a pET15b vector (N-terminal 6xHis tag) and induction by isopropyl β-D-1-thiogalactopyranoside (IPTG). The resulting 6xHis-IN fusion protein was purified using Ni-NTA agarose (Qiagen, Couraboeuf, France) loaded on a Poly-prep chromatography column (Bio-Rad, Marnes-La-Coquette, France). Elution was performed with increasing imidazole concentrations (20, 60, 100, 250, 750 mM). Protein purity in each fraction was observed on a 10% denaturing SDS-PAGE gel with Coomassie Brilliant Blue R-250 staining. Fractions of interest were then dialyzed (Thermo Scientific, Courtaboeuf, France, Slide-A-Lyzer 10K MWCO) to remove imidazole (25 mM PIPES pH 6.8; 750 mM NaCl; 50 µM ZnCl_2_;.0.1 mM EDTA; 50% glycerol). Protein concentration was measured by spectrophotometry (Thermo Scientific, Nanodrop 2000) (molecular weight: 34 kDa, extinction coefficient: 61,420 L.mol^−1^.cm^−1^), and solutions were stored at −20 °C.

### 2.4. IN-GCN2 Interaction Assay

The interaction between IN and GCN2 was monitored using AlphaLISA technology (Revvity, Bussy Saint Martin, France). The reaction was performed in a shallow-well 384-well microplate (Revvity, AlphaPlate-384SW, ProxiPlate) with 24 nM GST-GCN2, 700 nM 6xHis-IN, 10 µg/mL of nickel chelate acceptor beads and glutathione donor beads, and the test compound or an equivalent amount of DMSO (solvent at 10% final concentration) in a buffer composed of 50 mM Tris-HCl pH 7.5, 1 mM DTT, 0.05% Tween 20, 0.1% bovine serum albumin (BSA), and 40 mM NaCl (final volume 15 µL). First, the reaction was initiated by adding the various components except the donor beads. After 20 min at room temperature, the donor beads were added and the plate sealed. Data acquisition was performed 120 min later using a Victor Nivo Multimode Plate Reader (Revvity). Means, standard deviations (SD), and graphical representation were generated using Prism 8.4.3 software (GraphPad, Boston MA, USA).

### 2.5. Phosphorylation Assay

An in vitro phosphorylation assay was performed by incubating the kinase GST-GCN2 (25 nM) with its substrate His-IN (700 nM) in a reaction buffer composed of 15 mM MgCl_2_, 10 mM DTT, 10 mM Tris-HCl pH 8.0, 100 µM ATP, 7.4 mM MnCl_2_, 0.05% NP40, and 1.5 µCi ATP [γ-32P] (6000 Ci/mmol). The reaction mix was incubated at 30 °C for 60 min and stopped by the addition of Laemmli buffer (50 mM Tris pH 6.8, 2% SDS, 10% glycerol, 5% β-mercaptoethanol, 0.2% bromophenol blue). Samples were separated on a 10% denaturing SDS-PAGE gel, and proteins were stained using Coomassie Brilliant Blue R-250. Phosphorylation was revealed by autoradiography (Fujifilm, Montigny-le-Bretonneux, France, Imaging Plate BAS-MS) after overnight exposure and imaging on a FLA-5000 Imaging System (Fujifilm). Phosphorylation signals were quantified and analyzed using ImageQuant TL 10.1 analysis software (Cytiva, Velizy-Villacoublay, France) and Prism 8.4.3 software (GraphPad).

### 2.6. Strand Transfer Assay

IN ST activity was monitored using a previously described gel-based assay [27]. Briefly, 400 nM of IN and 20 nM of substrate were incubated in reaction buffer (50 mM MOPS pH 7.2, 7.5 mM MgCl_2_, 14 mM 2-mercaptoethanol), with the test compounds or an equivalent amount of DMSO. After 1 h at 37 °C, reactions were stopped by addition of one volume of loading buffer (95% formamide, 0.025% SDS, 1 mM EDTA) and products were separated using 16% sequencing polyacrylamide gels.

### 2.7. Cell-Based Assays

Compounds were tested for their cytotoxicity and antiviral properties using P4 MAGI CCR5+ cells (BEI resources, Manassas VA, USA; ARP-3580). Cells were plated in 96-well plates at a density of 10,000 cells per well and allowed to adhere for 24 h. Serial dilutions of compounds or an equivalent amount of DMSO were added to the cells (DMSO final concentration 0.5%). Cells were either left as it for 24 h to measure cytotoxicity using CellTiter 96^®^ AQueous (per manufacturer’s instructions, Promega, Charbonnieres, France) or infected using replication-competent HIV-1_Laï_ (produced in house). After 24 h, cells were washed 3 times in PBS 1× and HIV replication was monitored using a fluorogenic assay by adding the reaction buffer (50 mM Tris–HCl pH 8.5, 100 mM β-mercaptoethanol, 0.05% Triton X-100, and 5 mM 4-methylumbelliferyl-B-D-galactoside (4-MUG)), incubating for 4 h at 37 °C, and acquiring fluorescence in a Victor Nivo multimode microplate reader (Revvity, excitation/emission wavelengths of 360/460 nm).

## 3. Results

### 3.1. Screening of Natural Products for IN-GCN2 Interaction Modulation

**Figure 1:**
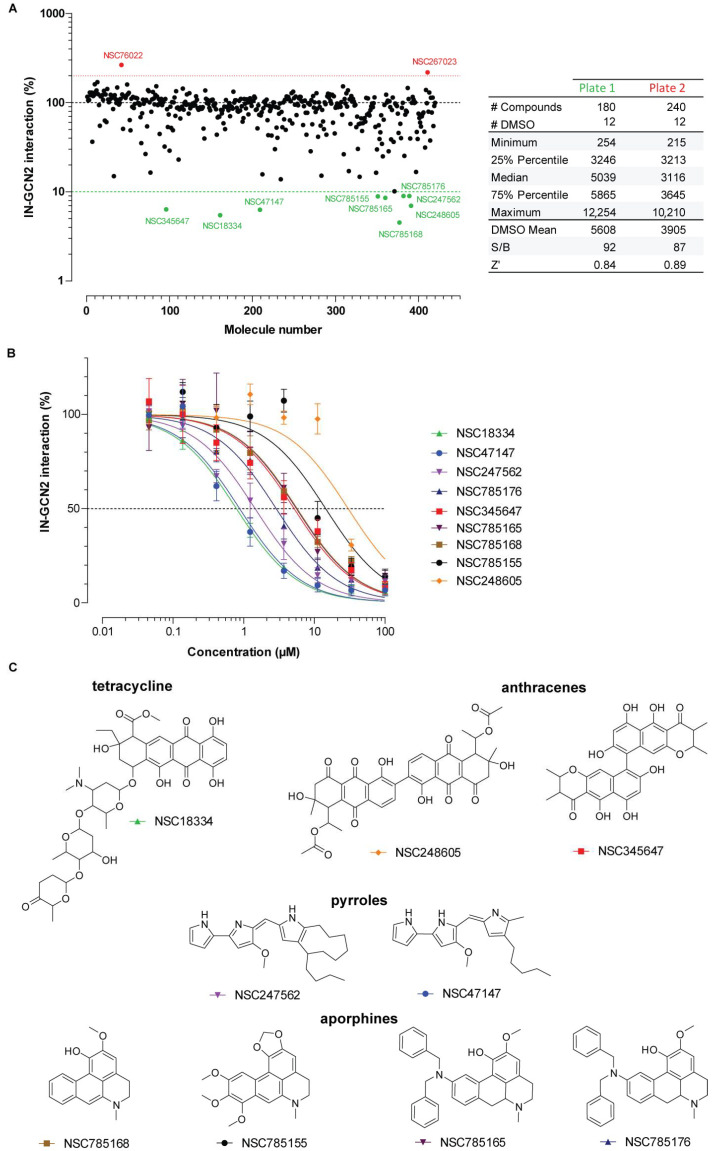
Screening of the DTP Natural Products Set. (**A**) Effect of natural extracts on the IN-GCN2 interaction measured by AlphaLISA. Each compound was tested at a unique dose of 100 µM in a single replicate. Signals were normalized to the DMSO control. Thresholds were set at 10% signal (green dashed line) to select molecules inhibiting at least 90% of the control signal (green dots) and 200% (red dotted line) to select potential stimulators (red dots). # = number. (**B**) Dose–response curves of the nine inhibitors of IN-GCN2 interaction. Mean and standard deviation were calculated from three replicates in three independent experiments (*n* = 9). The dashed line corresponds to 50% of the control signal. (**C**) Chemical structures of selected inhibitors.

In the present study, we used the Natural Products Set (420 molecules) from the National Cancer Institute Developmental Therapeutics Program (NCI DTP) to screen for modulators of the IN-GCN2 interaction in vitro. Each molecule was tested at a single dose (100 µM final concentration) and compared to the DMSO control (Figure 1A). The experiment was conducted on two separate AlphaPlate 384-shallow well plates, with 180 and 240 molecules respectively and 12 wells with DMSO as a control for each plate. In the control conditions, we obtained a mean signal of 5608 for plate 1 and 3905 for plate 2, which were considered the 100% values for the corresponding plate (Figure 1A). The standard deviation was ±856 for plate 1 and ±421 for plate 2. The mean background on each plate was 61 and 45 Alpha counts, respectively. The corresponding signal-to-background ratios were similar for the two plates, with a value around 90 (92 and 87, respectively, Figure 1A).

Based on the highest standard deviation of the two plates (15%, plate 1), the usual 3xSD threshold was not stringent enough and too many molecules would have been selected for validation (74 inhibitors below 55% residual interaction and 9 stimulators above 45% increase in signal). Thus, we arbitrarily selected a threshold at 10% residual signal (corresponding to 90% inhibition of the interaction, nine molecules) and at 200% (a 2-fold increase in signal, two molecules). Accordingly, only two compounds were considered as stimulators, NSC76022 and NSC267023, increasing the signal to 265.2% and 218.5% compared to DMSO, respectively (Supplementary Table S1). Regarding the inhibitors, NSC785168 was the most effective compound, with a more than 95% decrease in signal (4.5% residual signal compared to DMSO). Next, the compounds NSC18334, NSC47147, NSC345647, NSC248605, NSC785165, NSC785155, NSC247562, and NSC785176 inhibited the IN-GCN2 interaction with only 5.5 to 9% residual signal compared to the DMSO control. Of note, there was no apparent plate bias as two out of nine inhibitors (NSC18334 and NSC345647) and one out of two stimulators (NSC76022) were part of the first plate of the screen (containing 180 out of 420 tested compounds).

Altogether, eleven compounds were selected for dose–response assessment using concentrations ranging from 100 µM to 46 nM following a 3-fold serial dilution. Unfortunately, dose–response curves could not be obtained with the two stimulators. NSC267023 was found to be inactive while NSC76022 only exhibited an effect on IN-GCN2 interaction at the highest doses (with signals at 120% and 200% at 33 µM and 100 µM, respectively). Thus, only NSC76022 could be confirmed as a stimulator of IN-GCN2 interaction. However, due to the high dose required, we focused our attention towards molecules inhibiting the interaction.

IN contrast, all nine molecules were confirmed as inhibitors of the IN-GCN2 interaction (Figure 1B). Interestingly, the effect observed for each molecule at 100 µM is in agreement with the signal observed in the screen, with 86% to 94% inhibition (14% to 6% residual AlphaLISA signal). NSC248605 and NSC785155 were the least active molecules, with IC_50_ values of 30 µM ± 5 µM and 14.5 µM ± 2.5 µM, respectively. Next, NSC785168, NSC785165, and NSC345647 exhibited activity at similar concentrations, with IC_50s_ of 5.7 µM ± 0.6 µM, 5.6 µM ± 0.9 µM, and 5.2 µM ± 0.8µM, respectively. Then, NSC785176 and NSC247562 were active in the low micromolar range, with IC_50_ values of 2.9 µM ± 0.3 µM and 1.45 µM ± 0.2 µM, respectively. Finally, NSC47147 and NSC18334 were the most effective compounds, having sub-micromolar activities. Their respective IC_50_ values were 880 nM ± 110 nM and 790 nM ± 45 nM.

### 3.2. Chemotype Identification and Selection of Derivatives

The chemical structures of the nine molecules selected are presented in Figure 1C. Due to the aromatic nature of the selected molecules, their absorbance profile was determined; none of them absorbed at the wavelengths used by the AlphaLISA technology (λex 680 nm, λem 615 nm, Figure S1). The most active compound, NSC18334, is a tetracycline with a glycosidic side chain. Of note, two other tetracyclines were not selected but present in the DTP Natural Products Set. NSC265211 and NSC86005 inhibited the IN-GCN2 interaction but the residual signals of respectively 39.6% and 13.8% were above the selected threshold (Figure S2A and Table S1). Similarly, five other tetracycline molecules were included in the oncology set, NSC123127, NSC82151, NSC256439, NSC246131, and NSC256942, but none of them were selected at the time of the study (Figure S2B) [26].

Next, NSC248605 and NSC345647 are bianthracene derivatives, with NSC248605 presenting a comparable anthraquinone arrangement to that observed in NSC18334. Interestingly, a similar three-ring-based anthraquinone chemotype was selected, NSC279836, which was the most active compound of our pilot study [26].

NSC47147 and NSC247562 are pyrrole derivatives composed of three successive pyrrole rings and various aliphatic substitutions on one end of the molecule. Looking at the 550+ molecules tested (133 from the Oncology Set and 420 from the Natural Products Set), we could not find any other compound (active or inactive) that could be included in this family of molecules.

Lastly, NSC785168, NSC785155, NSC785176, and NSC785165 are aporphine derivatives with various substitutions. NSC785168 represents the simplest molecule, while NSC785155 has additional methoxyl groups and a dioxolane ring, NSC785165 and NSC785176 harbor a dibenzylamine substitution. Moreover, NSC785165 and NSC785176 only differ by their formulation, being mono- or di-hydrochloric acids, respectively. Accordingly, only one of them was kept for further analysis, NSC785176. Interestingly, nine other related molecules were present in the library but were not selected (Figure S3 and Table S1). NSC785154, NSC785178, NSC785158, NSC785171, and NSC785160 were inhibitors to a certain level with residual IN-GCN2 interaction signals of 16.4%, 16.7%, 25%, 25.3%, and 29.9%, respectively. NSC785159, NSC785170, and NSC36351 were inactive (within 10% of the DMSO control), while NSC266535 induced a slight stimulation of the interaction (39% increase in signal).

Altogether, four main chemotypes could be selected: tetracyclines (NSC18334), anthracenes (NSC345647, NSC248605), aporphines (NSC785168, NSC785155, NSC785176, NSC785165), and pyrroles (NSC47147, NSC247562). Accordingly, a homology search was performed in the PubChem database (substructure key-based 2D Tanimoto similarity) to look for derivatives of the eight molecules identified (except for NSC785165), along with NSC279836. The list obtained for each molecule was filtered to remove duplicates and to only keep compounds with an NSC number (thus, possibly available through the DTP). Altogether, we identified thousands of derivatives, hundreds included in the DTP and about 50 that were available for immediate testing.

### 3.3. Structure–Activity Relationship Studies

#### 3.3.1. Tetracyclines

**Figure 2:**
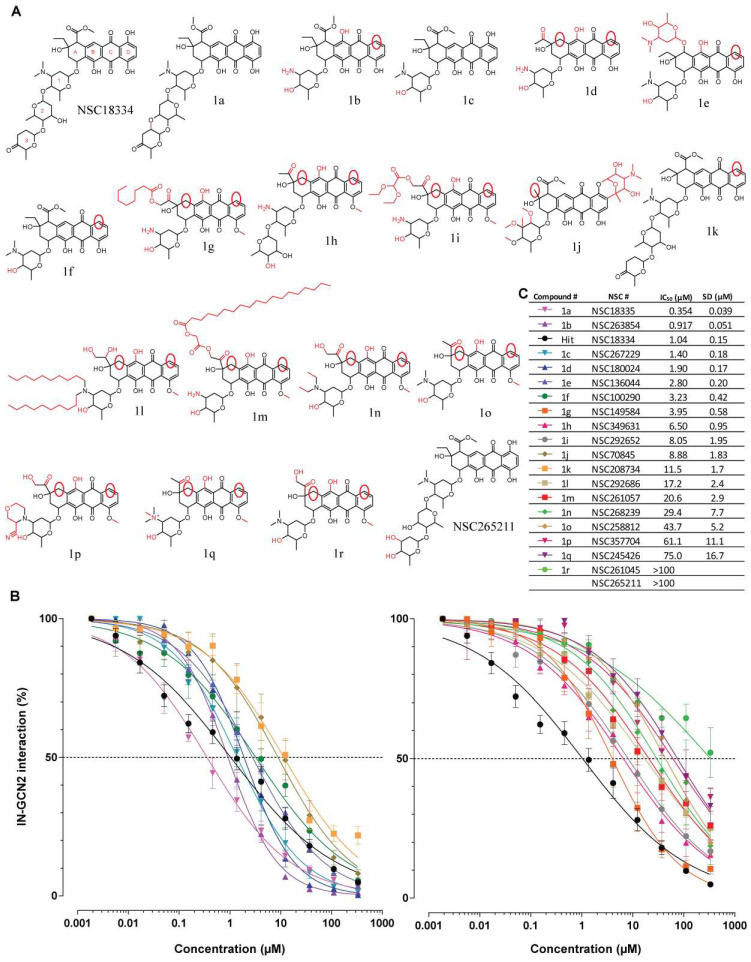
SAR study of tetracyclines. (**A**) Chemical structures of NSC18334 and its derivatives. Chemical modifications compared to NSC18334 are highlighted in red. (**B**,**C**) Inhibition of the IN-GCN2 interaction by tetracyclines compared to NSC18334. Dose–response curves and IC_50_ and SD values derived from at least three independent experiments. The dashed lines correspond to 50% of the control signal. # = number.

Starting from NSC18334 as a lead compound, we identified 18 derivatives available through the DTP (Figure 2A). Fresh dilutions of all 19 molecules from powder vials were tested in the IN-GCN2 interaction assay, and the corresponding IC_50_ values and standard deviations were calculated (Figure 2B). NSC18334 had a similar activity (1.04 µM ± 0.15 µM) compared to the plated compounds used in the screen (790 nM ± 45 nM, Figure 1). Interestingly, the derivatives exhibited a wide range of activity, with IC_50s_ from >300 µM to as low as 350 nM.

Compounds **1a** (NSC18335) and **1c** (NSC267229) harbor the same tetracycline core as present in NSC18334 (Figure 2A). Differences arise from the glycosidic chain, with **1a** having an additional cyclization of the triglycosidic moiety while 1c has a monoglycosyl. **1a** was the most active compound, with an IC_50_ of 354 nM ± 39 nM, corresponding to a 2.9-fold increase in activity compared to the parental compound NSC18334. **1c** was slightly less active than NSC18334, with an IC_50_ of 1.4 µM ± 0.2 µM. This seems to indicate that the presence of a monoglycoside is sufficient to inhibit the IN-GCN2 interaction, but locking the orientation of a longer chain (triglycoside) into a planar conformation could be beneficial. In addition, NSC265211 was related to NSC18334, with the presence of two hydroxyl groups on the third glycosyl group of the chain where NSC18334 has a carbonyl group (Figure 2A). The limited activity of NSC265211 compared to NSC18334 (about 60% inhibition at 100 µM, Table S1) highlights the important role of this third glycosyl group to engage specific interactions with the target.

Compound **1j** (NSC70845) harbors an extra bicyclic substitution fused on ring D of the tetracyclic moiety. The ethyl group of ring A is replaced by a methyl group. In addition, the single glycosyl substituent on ring A (similarly to **1c**) is substituted with two methyl and three methoxyl groups. This compound was still active in the IN-GCN2 interaction assay, but with an IC_50_ of 8.9 µM ± 1.8 µM, corresponding to an eight-fold decrease in potency compared to NSC18334 and a six-fold compared to **1c**. Interestingly, NSC86005, included in the Natural Products Set, harbored a similar modification of ring D (fused bicyclic) but lacked any substituent on ring A (no glycosidic chain, Figure S2). Even if it was only screened at a single dose, we found it similarly active against the IN-GCN2 interaction compared to **1j** (around 86% inhibition at 100 µM, Table S1). This tends to indicate that the presence of the glycosidic chain is not required for activity when in the presence of a bicyclic substitution fused to ring D.

Removing a hydroxyl group on ring D of NSC18334 leads to compound **1k** (NSC208734), which exhibited an IC_50_ of 11.5 µM ± 1.7 µM. This corresponded to an 11-fold decrease in activity compared to NSC18334, highlighting the importance of this substitution in the activity. However, a similar variation but in the context of **1c** (NSC267229) led to a more mitigated result. Compound **1f** (NSC100290) had an IC_50_ value of 3.2 µM ± 0.4 µM, corresponding to only a 2-fold decrease in activity compared to **1c**. Thus, it appeared that this hydroxyl group on ring D played a crucial role in the activity, depending on the length of the glycosidic chain.

Interestingly, compounds **1b** (NSC263854), **1d** (NSC180024), and **1e** (NSC136044) were derivatives also lacking the hydroxyl group on ring D but with an extra hydroxyl group on ring B. All three molecules were active, with IC_50_ values of 0.92 µM ± 0.05 µM, 1.9 µM ± 0.17 µM, and 2.8 µM ± 0.2 µM, respectively. Accordingly, **1b** is slightly more active than **1c** and in the range of NSC18334. Because **1b** lacks the dimethylamine substituent on the glycosyl group, we could not ascertain the importance of the presence of the extra hydroxyl group on ring B. However, compounds **1g** (NSC149584), **1h** (NSC349631), **1i** (NSC292652), **1l** (NSC292686), **1m** (NSC261057), **1n** (NSC268239), **1o** (NSC258812), **1p** (NSC357704), **1q** (NSC245426), and **1r** (NSC261045) all harbored the hydroxyl arrangement seen in **1b**/**1d**/**1e** but with a methoxyl group on ring D. They all exhibited a substantial increase in IC_50_, with values ranging from 4 µM for **1g** to >300 µM for **1r**. One piece of information could arise from previous data [26], where NSC82151 and NSC256439 inhibited the IN-GCN2 interaction at a similar level when tested at a single dose (around 70% inhibition at 100 µM). In fact, these two compounds derive from **1d** by the presence of a methoxyl group or a hydrogen in place of the hydroxyl on ring D. Thus, while the two molecules are less active than **1d** (~70% inhibition at 4 µM), it seems that the presence of a hydroxyl group is important at this position, either to create an H-bond with the target or to increase electron mobility throughout the tetracycline moiety.

#### 3.3.2. Anthracenes

**Figure 3:**
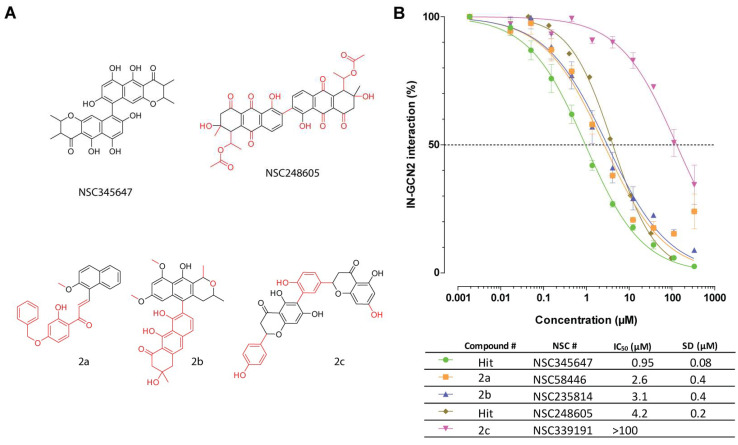
SAR study of anthracenes. (**A**) Chemical structures of NSC345647, NSC248605, and their derivatives. Chemical modifications compared to NSC345647 are highlighted in red. (**B**) Inhibition of the IN-GCN2 interaction by anthracyclines. Dose–response curves and IC_50_ and SD values derived from at least three independent experiments. The dashed line corresponds to 50% of the control signal. # = number.

For the anthracenes, only limited compounds with similarities to NSC345647 and NSC248605 were available (Figure 3A). Of note, fresh dilutions of NSC345647 and NSC248605 exhibited better activities than that observed during the initial screen from a plated source. They inhibited the IN-GCN2 interaction with IC_50_ values of 0.95 µM ± 0.08 µM and 4.2 µM ± 0.2 µM, respectively (Figure 3B). This corresponded to a 5- to 6-fold increase in potency. This apparent discrepancy may be due to a problem with the screening plate (prepared directly by the DTP) or a stability issue for the compound. Still, derivatives **2a** (NSC58446) and **2b** (NSC235814) were found to be active, with IC_50_ values in the single-digit micromolar range (2.6 µM ± 0.4 µM and 3.1 µM ± 0.4 µM, respectively), while **2c** (NSC339191) was almost inactive (>100 µM). Because of the limited number of molecules, no structure–activity relationship could be determined. Nonetheless, looking only at NSC345647, NSC248605, and **2b**, the three most alike molecules, it seems that the orientation of the two tricycles is not crucial in the overall activity of the molecule. While more in-depth analysis is required to better understand the mechanism of action, the constraint inherent with this single-bond linker might indicate that only one tricyclic moiety is used to bind its target.

**Figure 4:**
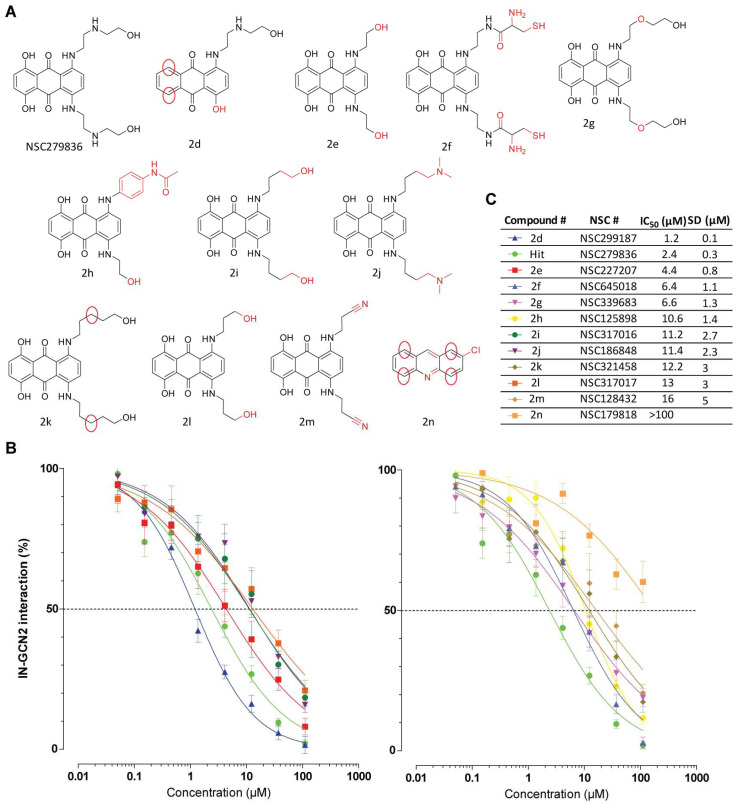
SAR study of anthraquinones. (**A**) Chemical structures of NSC279836 and its derivatives. Chemical modifications compared to NSC279836 are highlighted in red. (**B**,**C**) Inhibition of the IN-GCN2 interaction by anthraquinones compared to NSC279836. Dose–response curves and IC_50_ and SD values derived from at least three independent experiments. The dashed lines correspond to 50% of the control signal. # = number.

Additionally, NSC279836 is an anthraquinone previously identified for which we were able to select 11 derivatives (Figure 4A) [26]. Compound **2n** (NSC179818) is an acridine derivative that represents a simple version of the tricyclic core of NSC279836. The limited activity observed with **2n** (IC_50_ >100 µM) highlights the fact that the electron density and mobility of the anthracene core might be important, along with specific substitutions, to allow additional contact with the target. Interestingly, all other derivatives differed from NSC279836 only by the length and nature of the two aliphatic arms. Compounds **2e** (NSC227207), **2f** (NSC645018), **2g** (NSC339683), **2h** (NSC125898), **2i** (NSC317016), **2j** (NSC186848), **2k** (NSC321458), **2l** (NSC317017), and **2m** (NSC128432) were active in the low micromolar range, with IC_50_ values ranging from 4.4 µM to 16 µM (Figure 4B). The most striking difference related to the position of the H-bond donor/acceptor along the aliphatic chain. While they all presented a secondary amine at position 1, it appeared important to have either a secondary amine or alcohol at position 4 and to a lesser extent at position 7. This is also the case with compound **2d**, which exhibited increased activity compared to NSC279836, with an IC_50_ value of 1.2 µM ± 0.1 µM compared to 2.4 µM ± 0.3 µM. Even if this two-fold decrease in IC_50_ was moderate, it indicated that only one arm is sufficient for the activity of the molecule. As seen with the tetracyclines, the absence of two hydroxyl groups on ring A compared to NSC279836 may also be detrimental to the overall activity of **2d**.

#### 3.3.3. Aporphines

**Figure 5:**
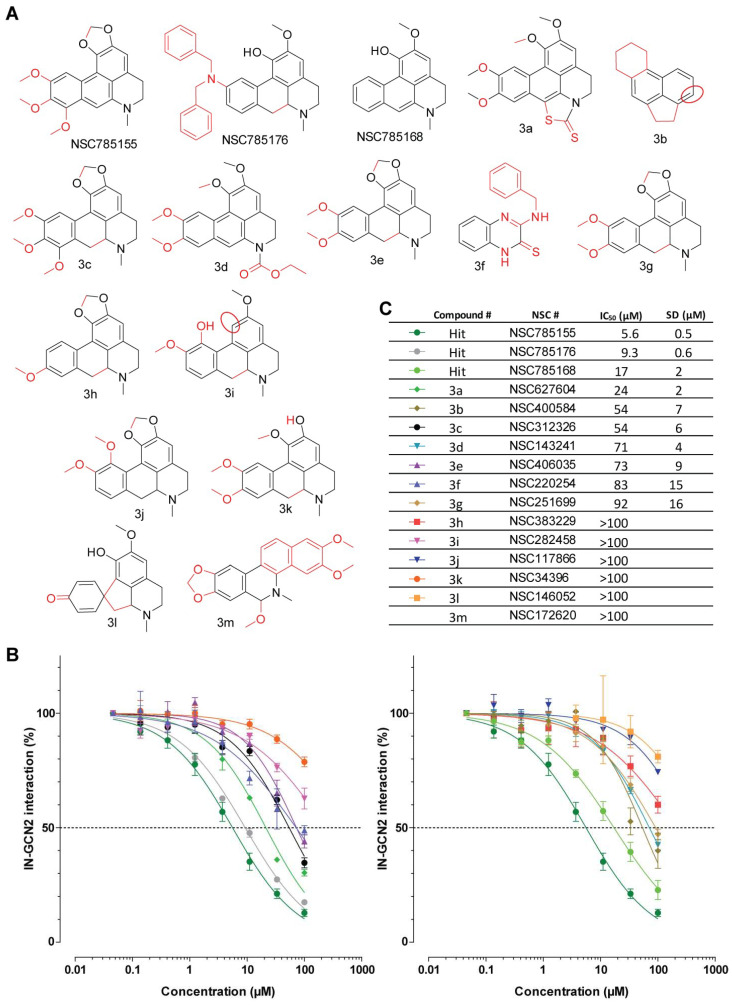
SAR study of aporphines. (**A**) Chemical structure of NSC785155, NSC785176, NSC785168, and their derivatives. Chemical modifications compared to NSC785168 are highlighted in red. (**B**,**C**) Inhibition of the IN-GCN2 interaction by aporphines compared to NSC785155. Dose–response curves and IC_50_ and SD values derived from at least three independent experiments. The dashed lines correspond to 50% of the control signal. # = number.

Alongside the three selected inhibitors NSC785155, NSC785176, and NSC785168, we tested 13 derivatives in the IN-GCN2 interaction assay (Figure 5A). Among those, six molecules were either inactive or had undetermined IC_50_ values (>100 µM), namely NSC383229, NSC282458, NSC117866, NSC34396, NSC146052, and NSC172620 (Figure 5B). For the rest of the derivatives, their IC_50_ values ranged from 24 µM to 92 µM. Accordingly, none were efficient inhibitors of the IN-GCN2 interaction and NSC785155 and NSC785176 were still the most active molecules. The main chemical difference between these two molecules is the closing of a dioxolane ring seen in NSC785155 and the presence of a dibenzylamine substitution on NSC785176. Both of them probably have a positive impact on the activity of the core structure represented by NSC785168. Indeed, NSC34396 was found to be inactive against the IN-GCN2 interaction, while the dioxolane-containing counterpart **3e** exhibited moderate inhibition (IC_50_ value of 73 µM ± 9 µM).

In contrast, the introduction of a thiazolidine thiocetone on the bottom of the molecule (**3a**, NSC627604) did not seem to improve the activity of the molecule compared to the previously identified NSC785154 (~80% and 93.6% inhibition at 100 µM, respectively). Even an ethyl carbamate substitution, as seen in **3d** (NSC143241), appeared to be detrimental, with only ~55% inhibition at 100 µM.

Another difference that arose from comparing all these structures was the planarity of the molecules. Globally, active compounds are predicted to be planar or only slightly out of plane (due to the piperidine ring), while molecules that are not planar (with distortion induced by the central ring) were less active to inactive. A first example of this possible dependency on the global conformation is the better activity observed with compound **3e** (NSC406035, S isomer) compared to **3g** (NSC251699, R isomer), which are stereoisomers. Similarly, the introduction of a single hydrogen atom in the central ring of the active compound NSC785168 is sufficient to abolish the activity of the corresponding derivative (NSC785170, Figure S3). Finally, **3c** (NSC312326) harbors an additional hydrogen atom on the aporphine core compared to NSC785155. Because of this unsaturated ring, 3D modeling of **3c** shows that the molecule adopts a slightly concave conformation. This non-planarity seemed to be highly detrimental for the activity, with a 10-fold decrease in activity compared to NSC785155.

### 3.4. Effect of Selected Molecules on the Activity of Recombinant Proteins In Vitro

**Figure 6:**
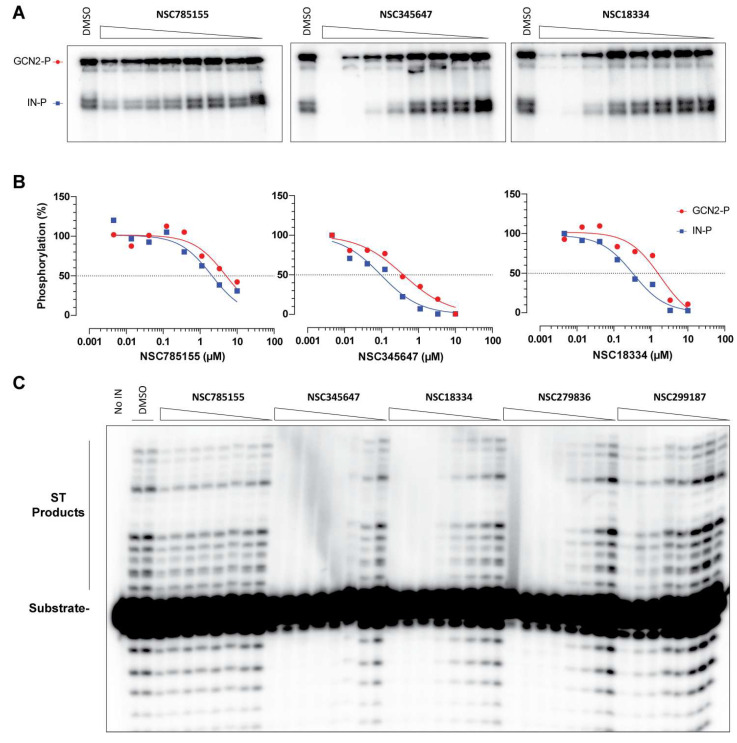
Effect of chemical modulators of the IN-GCN2 interaction. (**A**) Representative autoradiograph of a phosphorylation assay in the presence of NSC785155, NSC345647, and NSC18334. The phosphorylation of GCN2 and IN was monitored in the presence of DMSO or a decreasing concentration of compound (from 10 µM to 4.6 nM). (**B**) Inhibition of GCN2′s activation and IN’s phosphorylation by selected compounds. Gels were submitted to autoradiography and quantified. Means and non-linear regression are from two independent determinations. The dashed lines correspond to 50% of the control signal. (**C**) Representative autoradiograph of an ST gel-based assay. The left lane corresponds to the substrate DNA alone, followed by a duplicate control of IN in the presence of DMSO. Molecules are used from 333 µM to 152 nM following a 3-fold dilution, except NSC785155, whose concentration ranged from 111 µM to 51 nM.

To decipher the mode of action of these molecules in the inhibition of IN-GCN2 interaction, we tested a lead compound of each series for its activity in an in vitro phosphorylation assay—NSC345647 for anthraquinones, NSC18334 for tetracyclines, and NSC785155 for aporphines. In this assay, incubation of GCN2 (25 nM) with IN (700 nM) led to the autophosphorylation of GCN2 (Figure 6A, top band) and the phosphorylation of IN (bottom band). In our previous study, NSC279836 selectively inhibited the phosphorylation of IN, with an apparent IC_50_ in the low micromolar range, without affecting GCN2 autophosphorylation up to 300 µM [26]. Here, NSC785155 (aporphine) partially inhibited the phosphorylation of IN and had only a limited impact on the autophosphorylation of GCN2, even at concentrations necessary for the inhibition of the IN-GCN2 interaction (low micromolar, Figure 1 and Figure 6A,B). On contrast, full inhibition of IN phosphorylation was observed with NSC345647 and NSC18334 in the sub-micromolar concentration range (Figure 6A,B). Unlike NSC279836, the highest doses of NSC345647 and NSC18334 inhibited GCN2 autophosphorylation (Figure 6A). Still, a selectivity could be observed with the inhibition of GCN2 autophosphorylation, with this requiring about 5- to 10-times higher concentrations of the anthraquinone and tetracycline, respectively (Figure 6B). For all three compounds, the inhibition of IN phosphorylation and/or autophosphorylation of GCN2 was not due to protein aggregation (Figure S4).

Next, we investigated the impact of these molecules on the ST activity catalyzed by IN. Similar to the phosphorylation assay, NSC785155 was unable to fully inhibit ST even at 111 µM (Figure 6C). Again, NSC345647 and NSC18334 were potent inhibitors of ST activity with apparent IC_50_ values in the sub- and low-micromolar concentration ranges, respectively. Because NSC345647 is apparently the most potent inhibitor of ST activity, we wondered how it compared to NSC279836 and NSC299187 (**2d**), the lead compound of our previous study and its best derivative identified in this study. Interestingly, NSC279836 exhibited a two- to three-fold lower potency at inhibiting ST activity compared to NSC345647 (Figure 6C), which matches the difference in potency observed in the interaction assay (2.4 µM versus 0.95 µM, respectively, comparing Figure 3B and Figure 4C). However, NSC299187 (**2d**), which was more potent than NSC279836 at inhibiting IN-GCN2 interaction, actually lost potency at inhibiting IN compared to the parental compound (around 20-fold, Figure 6C). Moreover, NSC299187 (**2d**) tended to increase ST activity at lower concentrations (up to 170% compared to the DMSO control).

### 3.5. Biological Evaluation of Selected Compounds

Finally, we tested whether the identified molecules had an impact on HIV replication in a cell-based assay. Interestingly, we observed a wide range of cellular toxicity associated with this series of molecules (Table 1). While most molecules have little to no impact on cellular viability, some of them were toxic at very low concentrations (Figure S5A–E). Within the series of tetracyclines deriving from NSC18334, we observed a sub-micromolar toxicity for 9 out of 19 compounds. While already described in the literature [28], NSC357704 (**1p**) was toxic even at the lowest dose of about 2 nM used in this study (Figure S5C). In contrast, few molecules exhibited a cell growth stimulation (about 30–70% increase) within the sub- to low- micromolar concentration range. This was notably the case for NSC292652 (**1i**) and NSC247562 (Figure S5).

**Figure 7:**
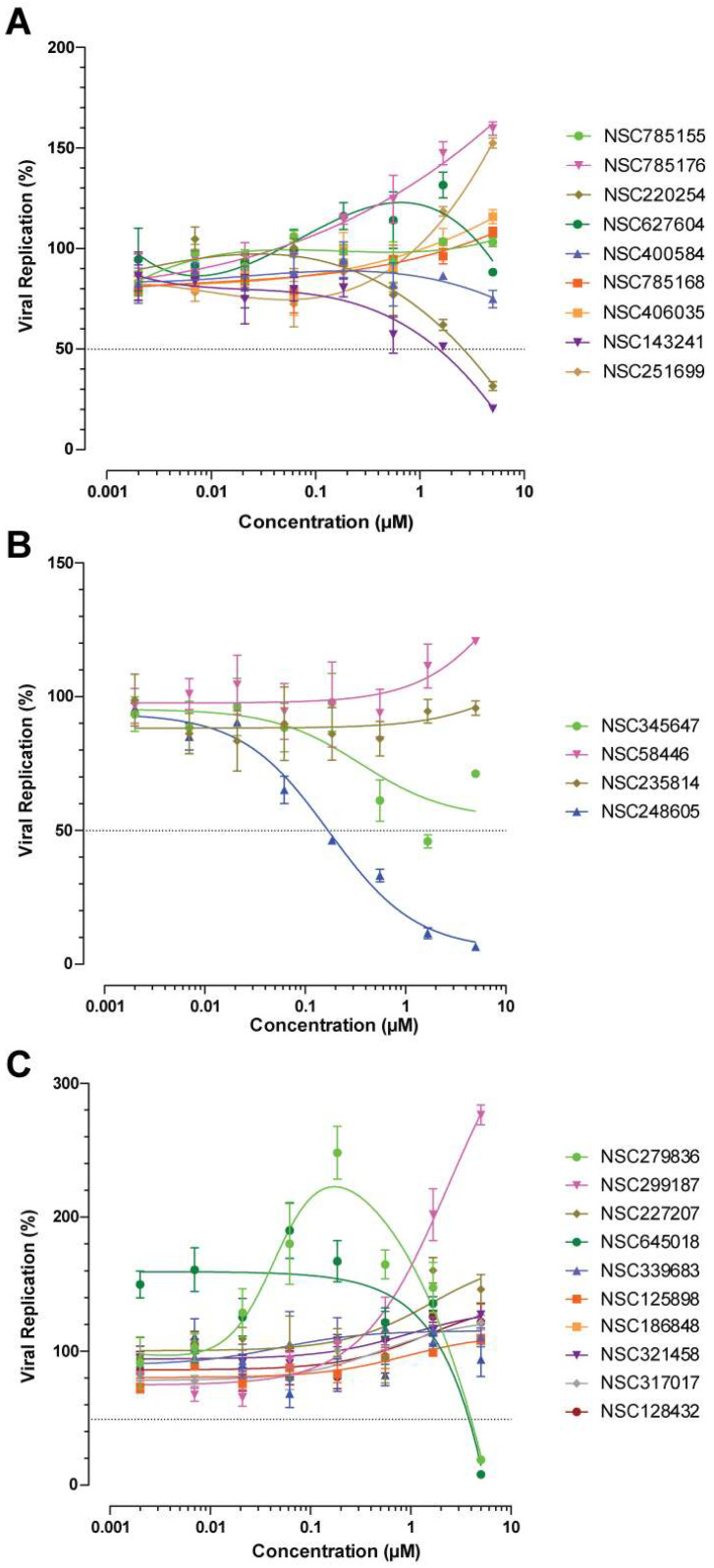
Effect of aporphine (**A**), anthracene (**B**), and anthraquinone derivatives (**C**) on HIV-1 replication. The dashed lines correspond to 50% of the control signal.

Selected molecules were then tested for their antiviral properties. Using a replication-competent HIV_Laï_ strain and MAGI reporter cells (harboring an integrated copy of the β-galactosidase gene under the control of the viral promotor), viral replication correlated with reporter activity. Accordingly, NSC143241 (**3d**) and NSC220254 (**3f**) were the only two aporphine derivatives that had an antiviral activity. Their EC_50_ values were 1 µM and 2.4 μM, respectively (Table 1 and Figure 7A). Conversely, NSC785176 and NSC251699 (**3g**) exhibited a pro-viral effect with a dose-dependent increase in viral replication of up to 150% (Figure 7A). To a lesser extent, a similar pattern could be observed with the anthracene derivatives (Figure 7B). NSC248605 and NSC58446 (**2a**) were anti- and pro-viral, respectively. Importantly, NSC248605 exhibited a good selectivity index of more than 30 with an antiviral activity in the nanomolar range (EC_50_ value of 167 nM ± 23 nM) without toxicity (CC_50_ value > 5 μM). Regarding the anthraquinone derivatives, only pro-viral activity was observed. Most compounds had a moderate effect, with 20–30% increased replication at the highest dose tested (Figure 7C). Still, NSC279836, NSC645018 (**2f**), and NSC299187 (**2d**) induced more pronounced increases. While the cellular toxicity exhibited by NSC279836 and NSC645018 (**2f**) quickly interfered with the increased replication, NSC299187 (**2d**) was non-toxic and a drastic increase in replication was observed, with up to 290% of the control replication in the presence of DMSO (Figure 7C). This is in agreement with the observed increase in ST activity using recombinant IN (Figure 6C).

## 4. Discussion

Because of the ability of HIV-1 to constantly evolve and adapt in its host, the emergence of resistance mutations affects all classes of antiretrovirals. New strategies aiming to target IN out of its active site should enable the resistance phenomenon with current INSTIs to be overcome. Its oligomeric state, post-translational modifications, and interactions with cellular or viral partners are all potential targets that can serve as a new platform for drug development. We previously demonstrated the role of GCN2 as a new partner of IN and the impact of this interaction and related phosphorylation on IN activities [25]. To date, there are only a limited number of inhibitors developed to target GCN2. The first to be described was A92, a triazolopyrimidine derivative, followed by GCN2iB, a pyrimidine derivative; both are ATP-competitive inhibitors [29].

The screening highlighted two pyrrole derivatives, namely NSC47147, also known as prodigiosin, and NSC247562, a cycloprodigiosin derivative (Figure 1B). Pyrrole derivatives have already been described in a large number of applications, including HIV IN inhibition [30]. Prodigiosin is a microbial secondary metabolite that has multiple cellular properties, including anticancer activity with low cytotoxicity on nonmalignant cells [31,32]. This molecule also showed antiviral potency against Herpes simplex virus and potential broad activity against enveloped viruses. It induces selective death of infected cells and targets signaling pathways in a way that is currently unclear [31,33]. To a certain extent, the prodigiosin highlighted in this study may adopt a similar spatial arrangement to that of A92, with its succession of three pyrrole groups. Prodigiosin has been linked to the ISR through the activation of endoplasmic reticulum stress and induction of the phosphorylation of eIF2α [32,34,35]. Together, these data suggest that prodigiosin may be inhibiting the IN-GCN2 interaction through targeting GCN2. In cells, both NSC compounds had a proviral effect at nontoxic concentrations (<200 nM, Figure S5). This is similar to the observed increased HIV-1 infectivity observed in the absence of GCN2 or when the phosphorylation of IN is blocked [25]. From its original tripyrrole arrangement, prodigiosin may serve as a flexible platform to design potent inhibitors of IN-GCN2 interaction (or GCN2 catalytic activity).

The second group, led by NSC785155, shares the core structure of aporphine alkaloids. These plant secondary metabolites are used in traditional medicine, with great effects in therapeutic investigations due to their antiplatelet aggregation, antispasmodic, anticancer, antimalarial, and antiviral activities, including anti-HIV activity [36,37,38,39,40]. Interestingly, Boustie and coworkers showed that NSC785158 (isoboldine) and NSC785178 (cassythivine), which were included in our study (Table S1), were active against poliovirus at a post-entry step during viral replication, with a selectivity index above 14 (compounds 7 and 9 in their study, respectively) [41]. SAR studies conducted on other aporphines and noraporphines exposed the fact that the spatial arrangement of the tetrahydroisoquinoline greatly impacted the activity of the compound [41]. In agreement with our data, the electron density and conformation of the aporphine moiety appear to be crucial in the capacity of the molecule to inhibit the IN-GCN2 interaction. Here, we found NSC785176 to increase viral replication at sub-micromolar concentrations, while NSC143241 (**3d**) had an opposite effect, with a decrease in viral replication (Figure 7A). The aporphine moiety appears to be a relevant starting point for the development of IN-GCN2 interaction modulators.

Next, we identified a group of polycyclic compounds with either a tri- or a tetracyclic arrangement. NSC18334 (cinerubine A) is an anthracycline extracted from bacteria of the genus *Streptomyces* spp. and considered among the most effective drugs to treat cancers, acting as intercalating agents and topoisomerase II poisons [42,43]. Here, we show that NSC18334 inhibited the ST activity catalyzed by IN (Figure 6C), which is in agreement with the fact that NSC261045 (**1r**) and another derivative NSC354646 (not used here) were previously reported as micromolar inhibitors of IN in vitro [44]. NSC18334 also impaired the phosphorylation of IN and autophosphorylation of GCN2, performed in the absence of DNA, meaning that this family of molecules could be targeting the IN-GCN2 interaction independently of their intercalating properties (Figure 6A). Notably, epirubicin is an isomer of doxorubicin (the gold standard of this anticancer family of drugs) but less cardiotoxic than doxorubicin. It was also more potent at inhibiting the IN-GCN2 interaction [26]. Herein, we identified 18 novel derivatives that presented a wide range of activity against IN-GCN2 interaction (Figure 2). Even if half of them were cytotoxic in the high- to low-nanomolar concentration range, we identified three new derivatives with sub-micromolar antiviral activity (Table 1). Namely, cinerubin A (NSC18334), cinerubin B (NSC18335, **1a**), and adriamycin octanoate (NSC149584, **1g**) had selectivity indexes of >6.3, 7.9, and >10.1, respectively. Thus, it appears possible to develop an inhibitor that has better affinity for the IN-GCN2 interaction without the drawbacks of canonical anthracycline derivatives.

The last group, led by NSC345647 (chaetochromin), comprises bianthracene derivatives. Some flavone derivatives like quinalizarin and purpurin have already been reported to inhibit HIV-1 IN in vitro [45]. Interestingly, two compounds tested in this study were already reported in the literature as IN inhibitors. NSC235814 (**2b**), also known as Karwinskia toxin T-544, inhibited both 3′-P and ST, with an IC_50_ of 21 µM [30]. NSC248605 was also reported as a non-selective inhibitor of IN activities, with an IC_50_ value of 140 µM [44]. Because INSTIs exhibit a selectivity for the inhibition of ST over 3′-P, one could postulate that these molecules act differently. Here, we show that NSC345647 is also a potent ST inhibitor with an IC_50_ in the mid-nanomolar concentration range (Figure 6B); however, it was inactive in a cellular context (Figure 7B). Still, another molecule of this series, NSC248605 (Julimycin B2), was antiviral without apparent toxicity, resulting in a selectivity index of more than 30 (Table 1). Accordingly, it is the most potent molecule of this work and represents a promising lead towards the development of a bioactive molecule targeting HIV replication.

Because of the similarity of these molecules to mitoxantrone (NSC279836), which was selected in our first screen [26], we decided to include anthraquinones in our study. Mitoxantrone is a synthetic derivative of anthracenedione that is a cytotoxic DNA intercalating agent with anti-HIV IN activity [46,47]. It has already been linked to the ISR, having roles in the induction of eIF2α phosphorylation and calreticuline translocation that are dependent on the presence of GCN2 [48,49,50]. It has also been reported to be an inhibitor of another serine/threonine kinase, PIM1 [51]. Moreover, compound **2g** (NSC339683) and two other mitoxantrone derivatives have been successfully used to resolve the crystal structure of PIM1 (PDBIDs 4RC2, 4RC3, and 4RC4). In those structures, the role of the aliphatic arms of mitoxantrone was evident, with an H-bond involving the fourth atom of the arm with the hydroxyl group of N172/D186 and D128/D131, respectively (see PDBID 4RC4 in complex with NSC645017). It also appeared that having a longer arm may slightly alter the binding pose of the molecule, being unable to contact N172/186 (see PDBID 4RC3 corresponding to compound **2g**). Altogether, these data are in agreement with what we observed for the inhibition of the IN-GCN2 interaction and suggest that mitoxantrone and its derivatives may be potent GCN2 inhibitors. Interestingly, compound **2d** (NSC299187), with its single arm, had similar activity to mitoxantrone in inhibiting the IN-GCN2 interaction (Figure 4C), but had lost the ability to inhibit the ST activity (about 20-fold decreased potency, Figure 6C). Moreover, NSC299187 has already been found to be active against *Borrelia burgdorferi* in the micromolar range (minimum inhibitory concentration of 10–20 µM) [52]. Thus, it may represent a good starting point to develop novel derivatives with increased activity and specificity over other kinases. Despite the fact that none of these molecules were toxic up to 1 µM (Table 1), none of them had antiviral activity either. Instead, we observed a general increase in viral replication, with NSC299187 being the most potent (up to a three-fold increase, Figure 7C). This could be in agreement with an observed effect where GCN2 depletion increases viral replication. Intriguingly, NSC279836 exhibited a dose-dependent hook effect that might correspond to an initial targeting of the IN-GCN2 interaction (or GCN2 activity) followed by a mix of toxicity and IN inhibition. Altogether, this series of compounds represents a good starting point towards the development of novel inhibitors.

Recently, more attention has been given to GCN2, mostly in the field of cancer research. Accordingly, more catalytic inhibitors have been developed [53,54]. Nevertheless, due to the recurrent limited selectivity for a kinase, one must be careful when trying to modulate the activity of cellular sensors like the ISR kinases. Furthermore, it has been shown that chemical inhibition of ISR kinases could actually activate the ISR pathway [55,56,57]. Because GCN2 is more complex than the other ISR kinases and uses its other domains for regulation, targeting the IN binding site instead of the ATP binding pocket/active site may represent a viable avenue to increase selectivity. Here, we selected compounds that are structurally different from the known GCN2 inhibitors: tetracycline, anthracene, and aporphine derivatives. These new chemotypes may be interesting platforms for the development of novel GCN2 inhibitors that might overcome the ISR activation induced by other inhibitors and be very helpful in gaining a better understanding of GCN2 and its regulation of HIV replication.

## Figures and Tables

**Table 1:** Antiviral activity and cytotoxicity of selected compounds. SI = selectivity index. ND = not determined. # = observed stimulation. Full dose–response curves can be found in Figure S5.

Compound #	NSC #	CC_50_		EC_50_		SI
		(µM)	SD	(µM)	SD	(CC_50_/EC_50_)
**1a**	NSC18335	>5		0.494	0.09	>10.1
1b	NSC263854	>5		ND		
Hit	NSC18334	>5		0.79	0.197	>6.3
1c	NSC267229	0.589	0.139	ND		
1d	NSC180024	0.04	0.012	ND		
1e	NSC136044	0.724	0.248	ND		
1f	NSC100290	2.352	0.519	1.393	0.462	1.7
1g	NSC149584	1.673	0.389	0.211	0.077	7.9
1h	NSC349631	>5		1.659	0.534	>3
1i	NSC292652	>5		>5		
1j	NSC70845	0.311	0.079	ND		
1k	NSC208734	0.114	0.031	ND		
1l	NSC292686	>5		ND		
1m	NSC261057	>5		ND		
1n	NSC268239	0.512	0.144	ND		
1o	NSC258812	0.256	0.053	ND		
1p	NSC357704	<0.002		ND		
1q	NSC245426	>5		>5		
1r	NSC261045	0.225	0.049	ND		
Hit	NSC345647	>5		>5		
2a	NSC58446	>5		#		
2b	NSC235814	>5		>5		
Hit	NSC248605	>5		0.167	0.023	>29.9
2c	NSC339191	>5		ND		
2d	NSC299187	>5		#		
Hit	NSC279836	>5		#		
2e	NSC227207	>5		>5		
2f	NSC645018	3.477	0.936	#		
2g	NSC339683	>5		>5		
2h	NSC125898	>5		>5		
2i	NSC317016	>5		>5		
2j	NSC186848	>5		#		
2k	NSC321458	>5		>5		
2l	NSC317017	>5		>5		
2m	NSC128432	>5		>5		
2n	NSC179818	>5		ND		
Hit	NSC785155	>5		>5		
Hit	NSC785165	>5		>5		
Hit	NSC785176	>5		#		
Hit	NSC785168	>5		>5		
3a	NSC627604	>5		#		
3b	NSC400584	>5		>5		
3c	NSC312326	>5		ND		
3d	NSC143241	>5		1.016	0.312	>4.9
3e	NSC406035	>5		>5		
3f	NSC220254	>5		2.367	0.487	>2.1
3g	NSC251699	>5		#		
3h	NSC383229	>5		ND		
3i	NSC282458	>5		>5		
3j	NSC117866	>5		ND		
3k	NSC34396	>5		ND		
3l	NSC146052	>5		ND		
3m	NSC172620	>5		ND		
Hit	NSC47147	1.956	0.388	#		
Hit	NSC247562	3.85	0.947	#		

## Data Availability

The original contributions presented in this study are included in the article. Further inquiries can be directed to the corresponding author.

## Supplementary Materials

The following supporting information can be downloaded at: https://www.mdpi.com/article/10.3390/v17081138/s1, Table S1: AlphaLISA signals obtained during the screen for all 420 compounds tested; Figure S1: Absorbance profile of selected molecules. Figure S2: Structure of additional tetracycline derivatives. Figure S3: Structure of additional aporphine derivatives. Figure S4: Effect of selected molecules of proteins solubility. Figure S5: Biological evaluation of IN-GCN2 chemical modulators.
