# Supplementary material for: Isolation of New Chemical Modulators of the Interaction Between HIV-1 Integrase and the Cellular Restriction Factor GCN2
Source: Viruses. 2025 Aug 20;17(8):1138. doi: 10.3390/v17081138 (PMC12390684; doi:10.3390/v17081138)
Supplement: Supplementary file 1 [file viruses-17-01138-s001.zip › viruses-3356293-supplementary/suppl/viruses-3356293-supplementary-R2.pdf]

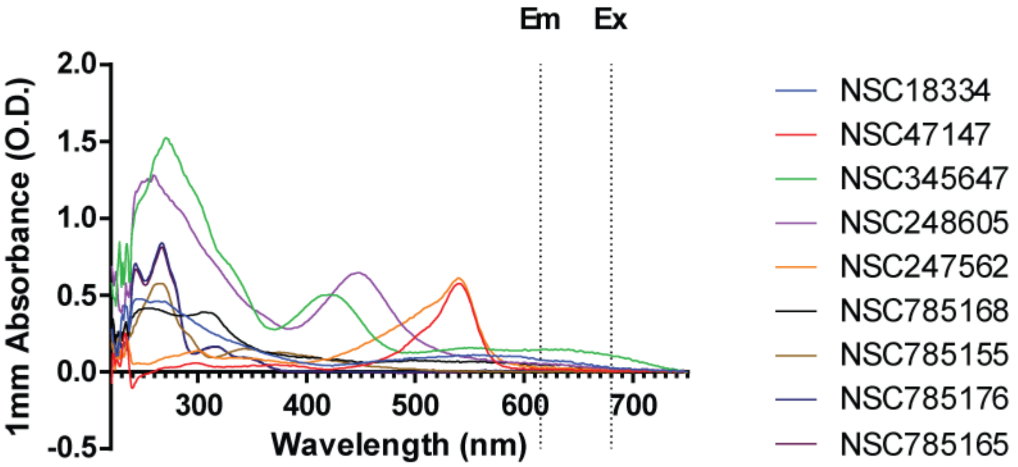

Figure S1. Absorbance profile of selected molecules. Absorbance of NSC compounds at a concentration of 1mM was measured from 220 to 750 nm (optical distance = 1 mm, step = 1 nm) using a nanodrop 2000 (ThermoFisher).

A

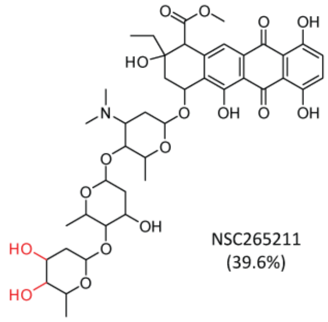

NSC265211
(39.6%)

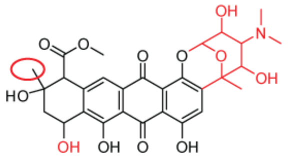

NSC86005
(13.8%)

B

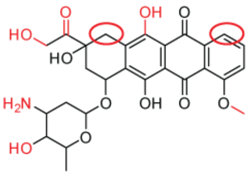

NSC256942

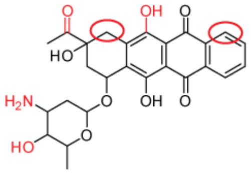

NSC256439

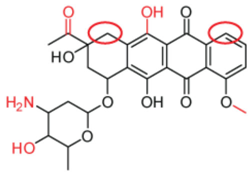

NSC82151

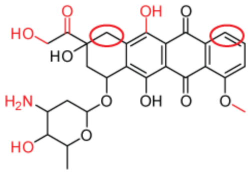

NSC123127

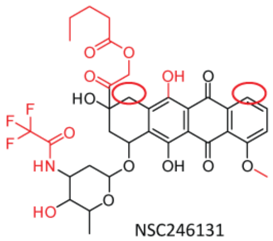

NSC246131

Figure S2. Chemical structure of non-selected tetracyclines. **A.** tetracyclines present in the natural products set. **B.** Tetracyclines present in the oncology set [24].

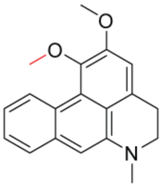

NSC785154
(16.4%)

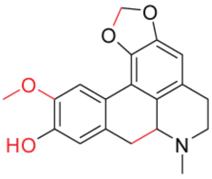

NSC785178
(16.7%)

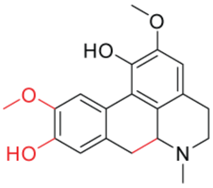

NSC785158
(25%)

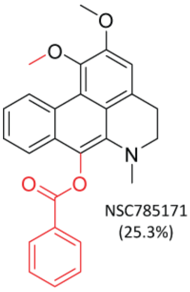

NSC785171
(25.3%)

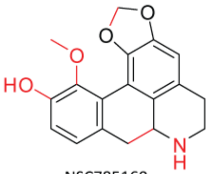

NSC785160
(29.9%)

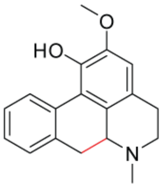

NSC785159
(94.2%)

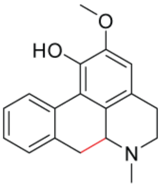

NSC785170
(99.5%)

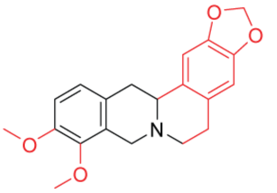

NSC36351
(108.6%)

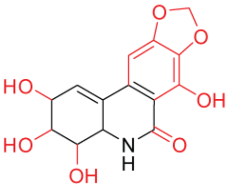

NSC266535
(139%)

Figure S3. Chemical structure of aporphines and derivatives present in the natural products set but non-selected during the screen.

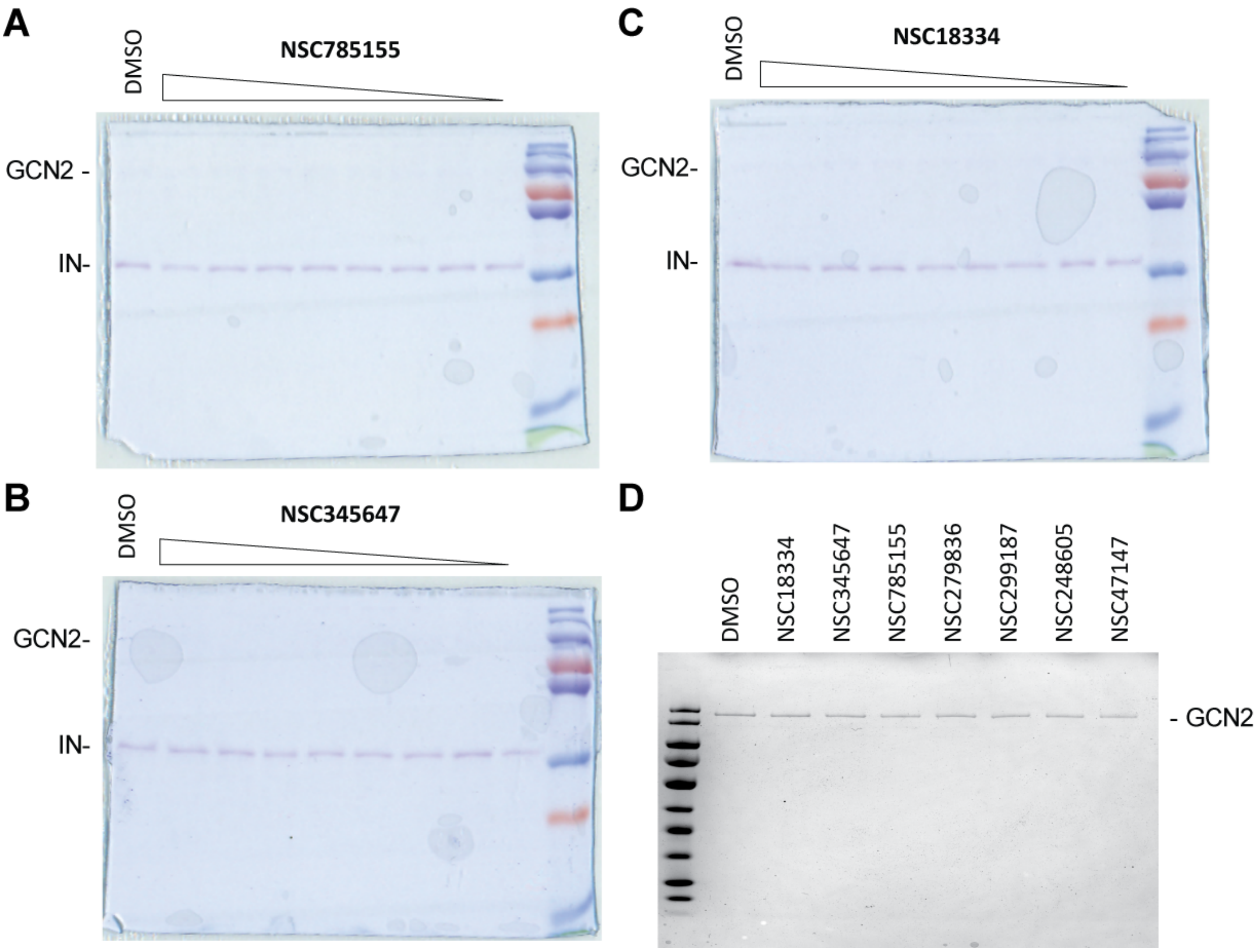

Figure S4. Effect of selected molecules of proteins solubility. Representative SDS-PAGE of a phosphorylation assay in the presence of NSC785155 (**A**), NSC345647 (**B**) and NSC18334 (**C**). Gels were colored with Coomassie Brillant Blue R-250 (Bio-Rad) to reveal the presence of GCN2 (25 nM, faint band) and IN (700 nM). **D**. Solubility of GCN2 in the presence of selected compounds. GCN2 was incubated with 100 µM of molecules or an equivalent volume of DMSO for 30 minutes at room temperature. Following a high speed centrifugation (20 minutes at 15000g), the soluble fraction was submitted to SDS-PAGE and coloration.

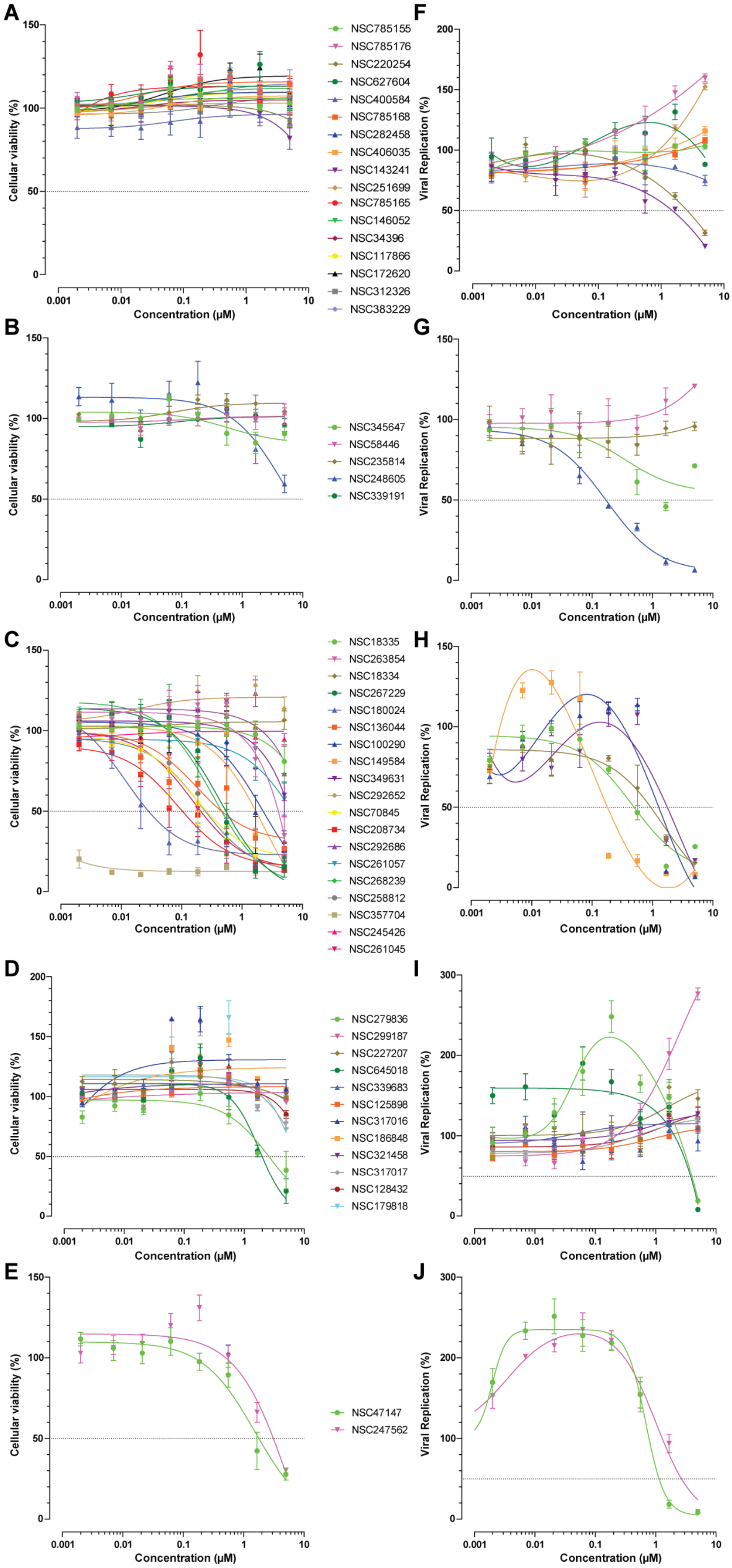

Figure S5. Biological evaluation of IN-GCN2 chemical modulators. **A-E.** Cellular viability of selected compounds measured using a formazan-based assay. **F-J.** Effect of selected compounds on HIV-1 replication using an LTR driven β-galactosidase reporter system.
